# Leukemia relapse following unmanipulated haploidentical transplantation: a risk factor analysis on behalf of the ALWP of the EBMT

**DOI:** 10.1186/s13045-019-0751-4

**Published:** 2019-07-04

**Authors:** Simona Piemontese, Ariane Boumendil, Myriam Labopin, Christoph Schmid, Fabio Ciceri, William Arcese, Yener Koc, Zafar Gulbas, Johanna Tischer, Benedetto Bruno, Depei Wu, Didier Blaise, Dietrich Beelen, Giuseppe Irrera, Annalisa Ruggeri, Mohamed Houhou, Mohamad Mohty, Arnon Nagler, on behalf of the Acute Leukemia Working Party of the European Society for Blood and Marrow Transplantation (EBMT)

**Affiliations:** 1 Hematology and Bone Marrow Transplant Unit, San Raffaele Scientific Institute, Milan, Italy; 2 Service d’Hématologie et Thérapie Cellulaire, Hôpital Saint Antoine, AP-HP, Paris, France; 3 INSERM, UMRs 938, Paris, France; 4 Universitätsklinik Augsburg, II Medizinische Klinik, Augsburg, Germany; 5 Hematology Division-Stem Cell Transplant Unit, University of Rome Tor Vergata, Rome, Italy; 6 Stem Cell Transplant Unit, Medical Park Hospitals, Antalya, Turkey; 7 Bone Marrow Transplantation Department, Anadolu Medical Center Hospital, Kocaeli, Turkey; 8 LMU-University Hospital of Munich-Grosshadern, Medizinischen Klinik III, Munich, Germany; 9 A.O.U Citta della Salute e della Scienza di Torino, Presidio Molinette, Torino, Italy; 10 First Affiliated Hospital of Soochow University, Suzhou, 215006 Jiangsu China; 11 Programme de Transplantation & Therapie Cellulaire, Institut Paoli Calmettes, Marseille, France; 12 Dept. of Bone Marrow Transplantation, University Hospital, 45122 Essen, Germany; 13 Centro Unico Regionale Trapianti, Alberto Neri, Bianchi-Melacrino-Morelli, Reggio Calabria, Italy; 14 Department of Pediatric Hematology and Oncology, IRCCS Bambino Gesù Children’s Hospital, 00165 Roma, Italy; 15 Université Pierre et Marie Curie, Paris, France; 16 Hematology and Bone Marrow Transplantation Division, Chaim Sheba Medical Center, Tel-Hashomer, Israel; 17 Sackler School of Medicine, Tel Aviv University, Tel Aviv, Israel; 18 ALWP office, Hopital Saint Antoine, Paris, France; 19 University Vita-Salute San Raffaele, Milan, Israel

**Keywords:** Leukemia relapse, Survival after relapse

## Abstract

**Background:**

As information on incidence, risk factors, and outcome of acute leukemia (AL) relapse after unmanipulated haploidentical stem cell transplantation (haplo-SCT) is scarce, a retrospective registry study was performed by the Acute Leukemia Working Party of the European Society for Blood and Marrow Transplantation.

**Methods:**

Among 1652 transplants performed for lymphoblastic and myeloid AL between 2007 and 2014, 587 patients (acute lymphoblastic leukemia (ALL) 131, acute myeloid leukemia (AML) 456) with detailed information were analyzed aiming to identify risk factors for post-transplant relapse and for overall survival (OS) after relapse.

**Results:**

The cumulative incidence of relapse at 3 years was 44% (35–53%) for ALL and 32% (27–36%) for AML (*p* = 0.023). In ALL, risk factors for relapse were disease status different from the first complete remission (CR1) at haplo-SCT (CR2 vs CR1: HR 2.85, *p* = 0.011; advanced vs CR1: HR 14.28, *p* < 0.0001) and male donor gender (HR 3.64, *p* = 0.0002), while in AML, risk factors were advanced disease at haplo-SCT (advanced vs CR1: HR 3.95, *p* < 0.0001) and comorbidities (HCT-CI) ≥ 3 (HR 1.75, *p* = 0.014). Transplants performed in more recent years were associated with lower relapse incidence (RI) in AML, but not in ALL (HR 0.91, *p* = 0.042). After relapse, median follow-up was 13 months (mos). OS at 1-year post relapse was 18%. Prognostic factors for superior OS after relapse were remission at time of haplo-SCT (CR vs advanced: HR 0.71, *p* = 0.028), time from transplant to relapse (≥ 5 mos vs < 5 mos: HR 0.530, *p* < 0.0001), and bone marrow as a stem cell source (peripheral blood (PB) vs bone marrow (BM): HR 1.473, *p* = 0.016).

**Conclusions:**

Risk factors for relapse after haploidentical transplantation were disease specific. Longer OS after relapse was achieved in particular by patients both in CR at haplo-SCT and relapsing more than 5 months after transplant (1-year OS 33%).

**Electronic supplementary material:**

The online version of this article (10.1186/s13045-019-0751-4) contains supplementary material, which is available to authorized users.

## Introduction

Allogeneic stem cell transplantation (allo-SCT) represents a curative option for intermediate- and high-risk adult acute leukemias (AL). In the absence of a matched donor, haploidentical donors are increasingly used with favorable outcomes [1–5]. Despite substantial improvements in non-relapse mortality (NRM) over the years, relapse of acute leukemia remains the leading cause of failure after allo-SCT. Following haploidentical transplant (haplo-SCT), relapse incidence (RI) ranges from 22 to 30% [6–9] up to 58% when reduced intensity conditioning (RIC) regimens are used [10].

In the haplo-SCT setting, major human leukocyte antigen (HLA) mismatches between donor cells and recipient leukemic cells may theoretically serve as potential targets for a strong allogeneic graft vs leukemia (GVL) effect. Nevertheless, recent studies from the Acute Leukemia Working Party (ALWP) of the European Society for Blood and Marrow Transplantation (EBMT) could only show a trend for a lower RI among patients with high-risk acute myeloid leukemia after haplo-SCT in comparison to identical sibling transplants [11, 12].

Several single-center-based studies have reported risk factors [13–15] and treatment of leukemia relapse after haplo-SCT [16–19]. Nevertheless, a systematic analysis aiming at identifying risk factors and timing of relapse, and describing possible treatments of leukemia relapse after haplo-SCT is still lacking. For these reasons, we decided to perform a large, registry-based study, using the EBMT-ALWP registry to identify risk factors and assessing outcome of leukemic relapse after haplo-SCT.

## Patients and methods

Inclusion criteria of the study were as follows: age ≥ 18 years; diagnosis of either acute myeloid (AML) or acute lymphoblasic leukemia (ALL) both de novo and secondary; non-ex vivo T cell-depleted SCT from a family donor with ≥ 2 HLA mismatches between donor and recipient; peripheral blood (PB) or bone marrow (BM) as source of SC; first allo-SCT (previous autologous SCT was allowed), regardless of stage at SCT. All patients underwent transplantation between January 2007 and December 2014.

Data for this retrospective multicenter analysis were provided and approved by the ALWP of the EBMT registry. The EBMT is a non-profit, scientific society representing more than 600 transplant centers mainly in Europe. The EBMT promotes all activity aiming to improve stem cell transplantation or cellular therapy, which includes registering all the activity relating to stem cell transplants. Data are entered, managed, and maintained in a central database with internet access; each EBMT center is represented in this database. There are no restrictions on centers for reporting data, except for those required by the law on patient consent, data confidentiality, and accuracy. Quality control measures included several independent systems: confirmation of validity of the entered data by the reporting team, selective comparison of the survey data with minimum essential data A (MED-A) data sets in the EBMT registry database, cross-checking with the National Registries, and regular in-house and external data audits. Since 1990, patients have provided informed consent authorizing the use of their personal information for research purposes.

Definitions and statistical analysis

Primary endpoints were [1] incidence and risk factors of leukemia relapse after transplant and [2] risk factors for overall survival (OS) after relapse. RI after transplant was defined as time from date of transplant to leukemia relapse. NRM was defined as death without evidence of relapse. Death without hematological relapse and relapse were competitive risks for relapse incidence (RI) and NRM, respectively. OS after relapse was defined as time from date of relapse and death from all causes. Acute GVHD was graded according to the modified Seattle Glucksberg criteria [20], and cGVHD according to the revised Seattle criteria [21].

Myeloablative conditioning regimens (MAC) were defined as including total body irradiation (TBI) > 8 Gy, or busulfan > 8 mg/kg, or more than one alkylating agent as per EBMT criteria [22].

The patient’s comorbidities were classified using the hematopoietic stem cell transplantation-specific comorbidity index (HCT-CI) according to Sorror et al [23].

The type I error rate was fixed at 0.05 for determination of factors associated with time to event. Multivariate analyses were performed using Cox regression models and stepwise regression procedures. Analyses were performed using the R statistical software version 3.2.3 (R Development Core Team, Vienna, Austria).

As ALL and AML differ in biology, risk classification, induction treatment, and outcomes including relapse rates [6, 24], and also because of significant differences in patients’ characteristics (Table 1), we analyzed these two populations separately.

**Table 1.**
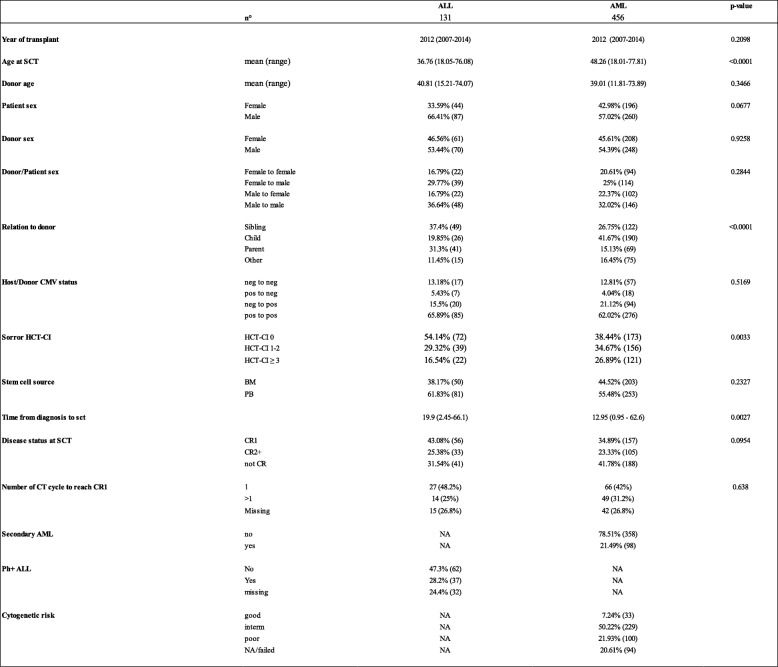
Patients’, donors’, and disease characteristics

*SCT* stem cell transplant, *CT* chemotherapy, *HCT-CT* hematopoietic cell transplantation comorbidity index, *CR* complete remission, *AML* acute myeloid leukemia, *Ph + ALL* Philadelphia positive chromosome acute lympholastic leukemia, *NA* not applicable

## Results

Patients’ and donors’ characteristics and overall outcome

**Fig. 1:**
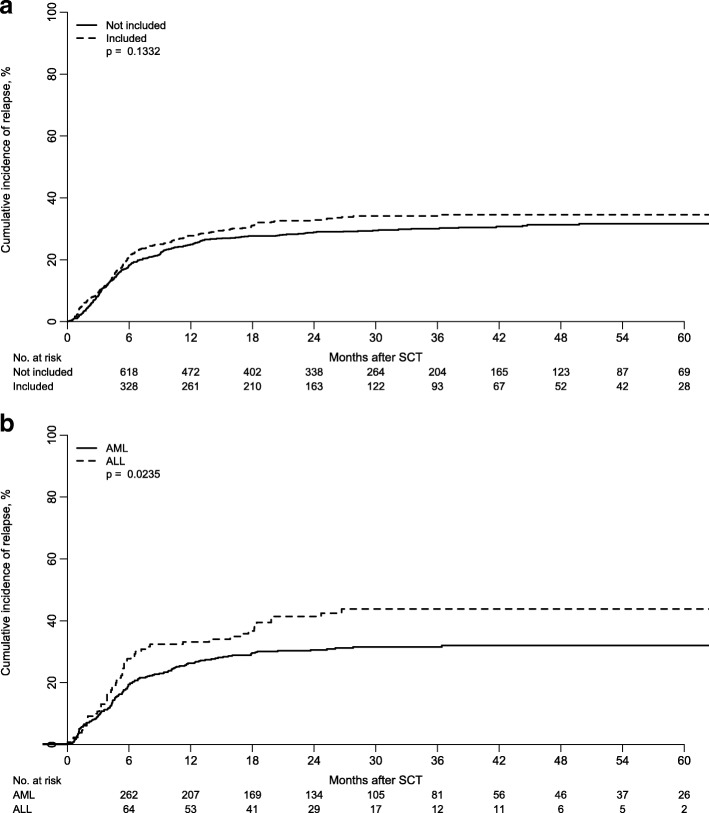
**a** Incidence of relapse in the 587 patients included in our analysis and the 1065 patients transplanted in the same years in the EBMT centers but not included in the final analysis due to incomplete data. **b** Incidence of relapse in patients with ALL and AML included in our analysis

Between January 2007 and December 2014, 1652 adult patients received a Haplo-SCT for acute leukemia. In 587 out of 1652 haplo-SCT (35%), detailed information on the characteristics of disease and management of relapse was provided by the respective transplant centers, allowing for the analysis of incidence and risk factors of leukemia relapse after transplant (RI) and overall survival (OS) after relapse. This group was well matched to the entire cohort of 1652 patients with respect to sex of patients, year of transplantation, source of stem cells, and diagnosis. In contrast, the patients reported more in detail were older, had been transplanted more frequently in advanced stage, and had been given more post-transplant cyclophosphamide (PT-Cy) for GVHD prophylaxis (Additional file 1: Table S1). Nevertheless, relapse incidence did not differ between the two groups (Fig. 1a), which is why we considered the cohort of patients reported more in detail as a representative.

Patients’ and disease characteristics of the 587 patients with detailed information are described in Table 1. Patients’ median age was 37 (18–76) years for ALL and 48 (18–78) years for AML (*p* < 0.0001). Conditioning regimens, graft vs host disease (GvHD) prophylaxis, and stem cell source are described in Table 2. Myeloablative conditioning regimens were mostly employed in ALL (92 patients, 70.23%) than in AML (221 patients, 48.46%) (*p* < 0.001). No statistical differences were found in means of in vivo T cell depletion (PT-Cy vs antithymocyte globulin (ATG)) or source of stem cells (PB vs BM) between the ALL and AML groups. ATG was used for in vivo T cell depletion in 46 patients with ALL (35.11%) and 169 with AML (37.06%) while post-transplant cyclophosphamide (PT-Cy) was applied in 85 ALL (64.89%) and 87 AML (62.94%) patients, respectively (*p* = 0.76). Peripheral blood stem cells were the stem cell source in 81 ALL (61.83%) and 253 AML (55.48%) patients, respectively (*p* = 0.233).

**Table 2.**
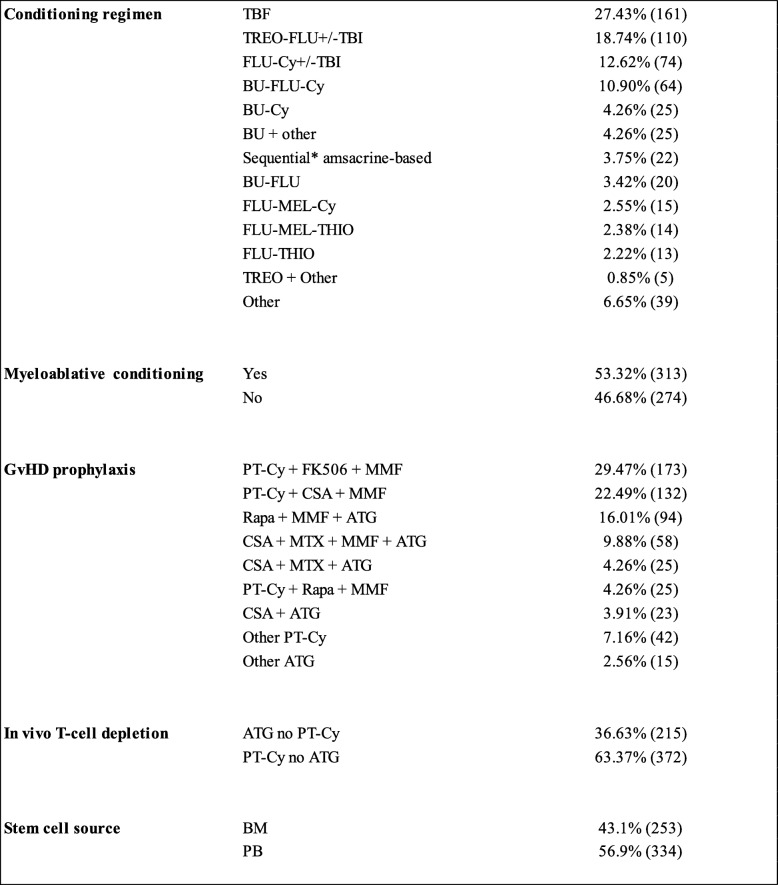
Conditioning regimen for haplo-SCT

*TBF* thiotepa–busulfan–fludarabine, *TREO* treosulfan, *FLU* fludarabine, *TBI* total body irradiation, *Cy* cyclophosphamide, *BU* busulfan, *MEL* melphalan, *THIO* thiotepa, *PT-Cy* post-transplant cyclophosphamide, *FK506* tacrolimus, *MMF* micophenolate mofetil, *Rapa* rapamycin, *ATG* antithymocyte globulin, *CSA* cyclosporine

^*^Sequential regimen of chemotherapy

### Relapse incidence after haplo-SCT

Relapse occurred in 197 out of 587 patients, 54 ALL (27%) and 143 AML (73%). Characteristics of relapsed patients are described in Additional file 2: Table S2. The CI of relapse at 3 years was 44% (35–53%) for ALL and 32% (27–36%) for AML (*p* = 0.023) (Fig. 1b).

Results for univariate analysis of risk factors for RI are shown in Additional file 3: Table S3. Among ALL patients, multivariate analysis revealed a lower RI after allo-SCT performed in CR1 (CR1 22%, CR2 48%, *p* < 0.001; CR ≥ 2 vs CR1: HR 2.98, *p* = 0.0087; not CR vs CR1: HR 14.83, *p* < 0.0001). Among ALL patients with advanced disease at transplantation, all but one experienced relapse. In addition, male donors were associated with higher RI (male vs female: HR 3.64, *p* = 0.0002). Risk factors for a higher RI AML were disease status at transplant other than CR (not CR vs CR1: HR 3.95, *p* < 0.0001), year of transplant (1-year increase: HR 0.91, *p* = 0.0425), and HCT-CI ≥ 3 (HCT-C ≥ 3 vs 0: HR 1.75, *p* = 0.0143) (Table 3).

**Table 3.**
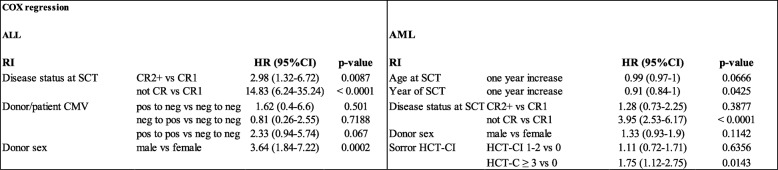
Multivariate analysis for relapse incidence after haplo-SCT for ALL and AML

*ALL* acute lymphoblastic leukemia, *AML* acute myeloid leukemia, *RI* relapse incidence, *HR* hazard ratio, *SCT* stem cell transplant, *CR* complete remission, *HCT-CI* hematopoietic cell transplantation comorbidity index, *CMV* cytomegalovirus

Characteristics and treatment of leukemia relapse post haplo-SCT

Median time from transplant to relapse was 4.91 months (0.03–62.77). Twenty-nine out of 197 (14%) relapsed patients experienced extramedullary relapse (EMR), 14 (26%) among ALL patients and 15 (10%) among patients with AML. EMR showed a trend for a later onset after transplant than hematological relapse (6.1 vs 4.9 months) (*p* = 0.08).

Treatment of relapse varied and included withdrawal of immunosuppression, chemotherapy, tyrosine kinase inhibitors (TKI), donor lymphocyte infusion (DLI), and a second allo-SCT (Table 4). Strategies were used either alone or in combination.

**Table 4.**
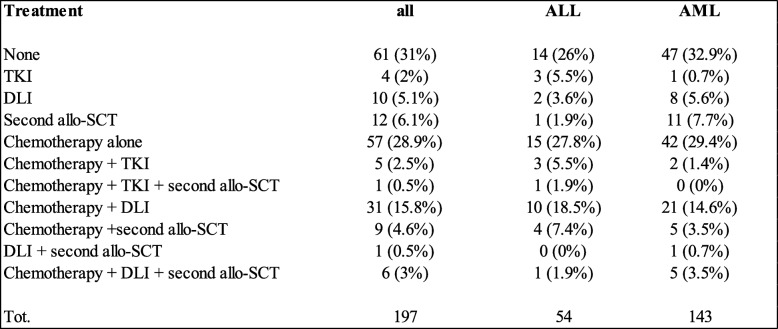
Treatment of relapse after haplo-SCT

*ALL* acute lymphoblastic leukemia, *AML* acute myeloid leukemia, *SCT* stem cell transplant, *TKI* tyrosine kinase inhibitors, *DLI* donor lymphocyte infusion

Outcome and risk factors for survival after relapse

Median follow-up from date of relapse to last contact was 13 months. OS was 18% and 8% at 1 and 2-year post relapse, respectively. Leukemia was by far the leading cause of death (154 patients, 90%), followed by infections (13 patients, 7.4%). Two patients died from GvHD (1.1 %), while 3 (1.5%) from other transplant-related causes.

**Fig. 2:**
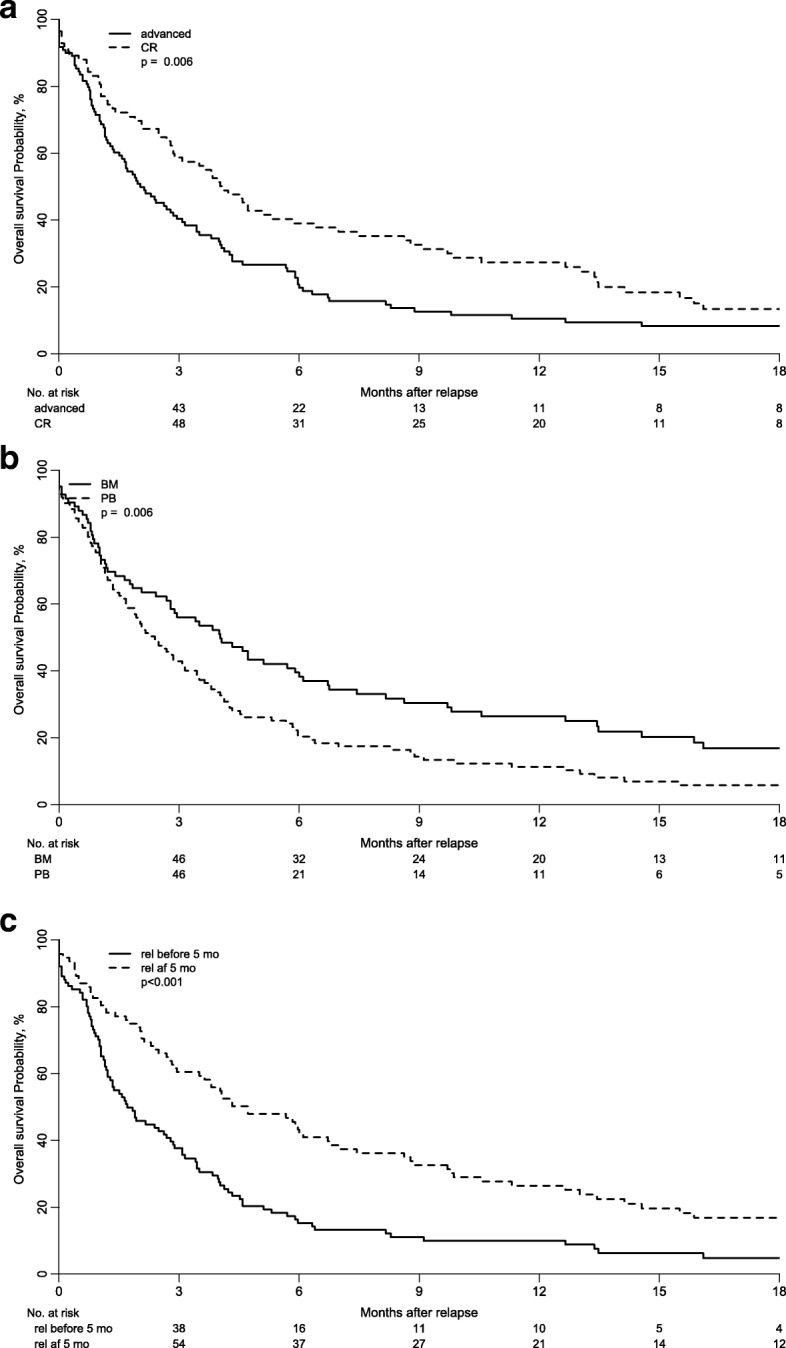
Overall survival from relapse according to **a** disease status at time of haplo-SCT, **b** stem cell source, and **c** time from transplant to relapse

The 1-year overall survival after relapse was higher for patients transplanted in remission (29% CR1, 25% CR ≥ 2, 10% not in remission, *p* = 0.023), for those with good/intermediate cytogenetic (25% good/intermediate vs 9% in those with poor/NA, *p* = 0.004), for patients who relapsed more than 5 months after haplo-SCT (26% > 5 months vs those who relapsed ≤ 5 months (10%, *p* > 0.0001)), in patients having received bone marrow grafts (26% BM, 11% PB, *p* = 0.006), and for patients without grade II–IV aGvHD (21% no, 6% yes, *p* = 0.09). The prognostic factors for higher OS confirmed by multivariate analysis were remission at time of haplo-SCT, time from transplant to relapse (> 5 months), and stem cell source (BM) (Table 5 and Fig. 2a–c). In 49 out of 197 patients (33%) that showed both remission at time of haplo-SCT and relapse > 5 months from transplantation, the 1- and 2-year OS were 33% and 14%, respectively (*p* = 0.003).

**Table 5.**
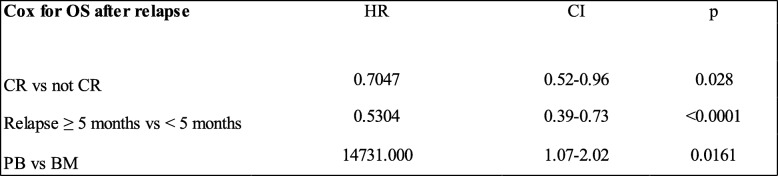
Multivariate analysis of risk factors for OS after relapse

*OS* overall survival, *HR* hazard ratio, *CI* confidence interval, *CR* complete remission, *PB* peripheral blood, *BM* bone marrow

Since the reason why patients have been assigned to the different treatments could not be evaluated retrospectively, no comparison among different strategies could be performed. Second allo-SCT was performed in 29 relapsing patients; out of them, 9 were in CR (≥ 2, 31%), while 20 were in relapse (69%) at the time of second haplo-SCT. The 1- and 2-year OS following the second allo-SCT were 25% (13–48%) and 12% (4–35%), respectively. Only 3 patients are alive at 6, 29, and 61 months from second allo-SCT.

## Discussion

Leukemia relapse is the leading cause of failure after allo-SCT including in transplants from haploidentical donors [1–5, 24]. Most studies comparing haploidentical donors to matched unrelated or related donors did not find any overall differences in terms of leukemia relapse according to donor type [6, 11, 12, 25, 26]. In contrast, Ciurea et al. compared haplo-SCT to transplants from unrelated donors in AML and described a higher incidence of relapse after haplo-SCT after RIC regimens [10], whereas Luo et al. reported a lower incidence of relapse in patients with hematological malignancies undergoing haploidentical versus matched sibling donor SCT in a setting of ATG-based immunosuppression [27].

The current report represents a large registry-study focusing on relapse after haplo-SCT for acute leukemia analyzing risk factors, timing, treatment strategies, and overall survival after relapse. In ALL, patients transplanted beyond CR1 had a higher RI after haplo-SCT. This finding is in line with extensive previous published literature [28–30] and confirm the importance of proceeding to transplant as soon as the patients is in CR1 for those patients with an indication for allo-SCT.

As part of registry-based study limitations, we were not able to study the impact of pre-transplant measurable residual disease on ALL relapse. Acute lymphoblastic leukemia immunophenotype was available only for a quarter of the patients so we could not analyze relapse incidence between B cell and T cell ALL. Nevertheless, in a previous publication from ALWP, Santoro et al. did not find any difference in haplo-SCT outcome between B-ALL and T-ALL [31].

Another risk factor for ALL relapse was donor gender; in particular, male donors were associated with a higher RI. As male donors were associated with a lower incidence of grade II–IV acute GvHD (aGvHD, Additional file 4: Table S4), one possible reason for the higher incidence of relapse could be a lower GVL effect. Recently, McCurdy et al. described a higher PFS after PT-Cy Haplo-SCT if grade II aGvHD had occurred [32]. Nakasone et al. [33] have previously reported lower incidence of relapse in a subset of male patients receiving allo-SCT from a female donor. Baron et al. [34] also reported a reduced incidence of acute leukemia relapse and a higher incidence of cGvHD in the combination of female donors to male patients. Of note, in our study, male donors were also a risk factor for a lower leukemia-free survival (LFS) and OS but not for higher NRM both in ALL and in AML.

Not unexpectedly, also in AML, disease status at transplant was a risk factor for higher relapse. In addition, patients with a HCT-CI ≥ 3 and transplanted in older age had a higher incidence of relapse. These findings might be explained by a lower conditioning intensity in elderly patients and those transplanted earlier or with higher comorbidities. However, the importance of the intensity of the conditioning regimens in preventing leukemia relapse after allo-SCT is debated. Rubio et al. had recently reported on behalf of the ALWP of the EBMT a trend for a higher incidence of relapse in patients with AML receiving a haplo-SCT with RIC regimen [35]. Ciurea et al. showed a similar 1-year incidence of relapse in AML patients after haplo-SCT using RIC and MAC regimens [10]. In our current study, the intensity of conditioning regimen did not affect RI neither in ALL nor in AML. Of note, RIC regimens were associated to a lower incidence of aGvHD II–IV in ALL and lower NRM in AML without affecting OS and LFS (Additional file 4: Table S4). Importantly, according to our data (Table 3), incidence of AML relapse post haplo-SCT is decreasing in transplants performed in more recent years. This might be in part due to modern approaches for the prevention of post-transplant relapse, including prophylactic or pre-emptive strategies such as hypomethylating agents, DLI, and anti-FLT3 inhibitors [36–39], although not enough data were available in the registry to study this aspect in detail.

Concerning stem cell source, Bashey et al. [40] have recently reported a lower incidence of relapse after haplo-SCT for acute leukemia using PBSC. In contrast, in our analysis, stem cell source did not affect RI neither in ALL nor in AML. Furthermore, bone marrow grafts were associated with a lower incidence of cGvHD in ALL patients without affecting OS, LFS, aGVHD, and NRM (Additional file 4: Table S4). Our current data are consistent with previous publication showing comparable results for PBSC or BM recipients in haplo-SCT [41].

Incidence of extramedullary relapse (EMR) was higher in our cohort comparing to previous reports (14% vs 4–10%). A possible explanation could be the higher number of ALL patients in active disease at time of transplant, a well-known factor associated with EMR [42–45]. In agreement with previously published literature, EMR relapses occurred later than hematological relapses [45] (6.1 vs 4.91 months), even if it was only a trend in our data. The exact cause of late transplant-failure at extramedullary sites is unclear but may result from the development of immune tolerance at peripheral sites (which are also shielded to a degree from chemotherapy and conditioning regimens) [46, 47].

It is well established that patients with acute leukemia relapsing after a first allograft have a poor prognosis with a survival of only few months [48, 49]. Chemotherapy-based re-induction may result in remission rates of up to 70%, but OS rarely exceeds 15–20% at 2 years [48, 49]. DLI can be an additional option, but patients with relapsed leukemia rarely experience long-term benefit [50].

In the current study, various treatment modalities were used to conquer the leukemia relapses post haplo-SCT with a limited success rate in agreement with previous publications, regardless of donor type [36, 51-52]. Similar to the report by McCurdy et al. [26], we observed an inverse correlation between time to relapse post haplo-SCT and outcome, with better 1-year OS in patients relapsing > 5 months post haplo-SCT. Further, haplo-SCT in CR was associated with improved outcome even after post-transplant relapse. Notably, we were able to identify a subgroup of patients with a better prognosis after relapse: Those 49 patients that were transplanted in remission and relapsed later than 5 months after transplant had better outcome and might be the most appropriate candidates for aggressive treatments such as second transplants. This approach is still intended to be curative; however, it is usually associated with higher toxicity and NRM [53–56].

Finally, stem cell source for the initial haplo-SCT influenced OS after relapse. Patients that received BM experienced a higher 1-year OS after relapse post haplo-SCT in comparison to those transplanted with PB (11% vs 2%). One explanation could be the slightly higher incidence of cGvHD, which is usually associated with GvL effects, among patients receiving PB (Additional file 4: Table S4, also shown by Bashey and colleagues [40]). This might indicate a kind of resistance against GvL effects in those patients developing relapse.

In summary, within the limitations of a retrospective registry study, this analysis underscores the importance of remission of disease at time of haplo-SCT and coordinated timing of transplants, aiming in both lowering relapse incidence and increasing survival among those patients who develop relapse anyway. Donor gender, patient comorbidities, and year of transplantation are additional prognostic factors for leukemia relapse. Overall survival after relapse is dismal, but we were able to identify a subgroup of patients with better prognosis, those transplanted in complete remission and relapsing > 5 months after haplo-SCT.

If confirmed in further studies, these findings may contribute to the early identification of patients at higher risk of relapse after haplo-SCT. Furthermore, they can help to overcome some of the risk factors for relapse in the pre-transplant setting or precociously planning post-transplant maintenance strategies. Finally, they can help us in selecting those patients that could better benefit from further treatments after relapse. Prospective trials comparing the different post-relapse therapeutic strategies may further help in dissecting the role of each treatment modality (Additional file 5: Table S5).

## Additional files

Additional file 1: **Table S1.** Characteristic of the 587 patients included in our analysis and the 1065 patients transplanted in the same years in the EBMT centres but not included in the final analysis due to incomplete data. (DOCX 19 kb)

Additional file 2: **Table S2.** Characteristics of relapsed patients. (DOCX 25 kb)

Additional file 3: **Table S3.** Univariate analysis for RI in ALL and AML. (DOCX 21 kb)

Additional file 4: **Table S4.** COX regression for ALL and AML. (DOCX 22 kb)

Additional file 5: **Table S5.** EBMT participating centers. (DOCX 20 kb)

## Data Availability

The datasets used and/or analyzed during the current study are available from the corresponding author on reasonable request.

## Abbreviations

AL: Acute leukemia
ALL: Acute lymphoblastic leukemia
ALWP: Acute Leukemia Working Party
AML: Acute myeloid leukemia
ATG: Antithymocyte globulin
BM: Bone marrow
CR: Complete remission
DLI: Donor leukocyte infusion
EBMT: European Society for Blood and Marrow Transplantation
EMR: Extramedullary relapse
GvHD: Graft vs host disease
GVL: Graft vs leukemia
HCT-CI: Hematopoietic cell transplantation-specific comorbidity index
HLA: Human leukocyte antigen
LFS: Leukemia-free survival
MAC: Myeloablative conditioning regimens
MED-A: Minimum essential data A
NRM: Non-relapse mortality
OS: Overall survival
PB: Peripheral blood
PT-Cy: Post-transplant cyclophosphamide
RI: Relapse incidence
RIC: Reduced-intensity conditioning
SCT: Stem cell transplantation
TBI: Total body irradiation
TKI: Tyrosine kinase inhibitors
