# Supplementary material for: Leukemia relapse following unmanipulated haploidentical transplantation: a risk factor analysis on behalf of the ALWP of the EBMT
Source: J Hematol Oncol. 2019 Jul 4;12:68. doi: 10.1186/s13045-019-0751-4 (PMC6610936; doi:10.1186/s13045-019-0751-4)
Supplement: Supplementary file 1 — Table S1. Characteristic of the 587 patients included in our analysis and the 1065 patients transplanted in the same years in the EBMT centres but not included in the final analysis due to incomplete data. (DOCX 19 kb) [file 13045_2019_751_MOESM1_ESM.docx]

**Additional file 1: Table S1. Comparison of patients from the ALWP registry who were and were not included into the present analysis**

SCT stem cell transplant; ALL acute lymphoblastic leukemia; AML acute myeloid leukemia; CR complete remission; PT-Cy post-transplant cyclophosphamide; aGvHD acute graft-versus-host disease; cGvHD chronic graft-versus-host disease
