# Supplementary material for: Leukemia relapse following unmanipulated haploidentical transplantation: a risk factor analysis on behalf of the ALWP of the EBMT
Source: J Hematol Oncol. 2019 Jul 4;12:68. doi: 10.1186/s13045-019-0751-4 (PMC6610936; doi:10.1186/s13045-019-0751-4)
Supplement: Supplementary file 3 — Table S3. Univariate analysis for RI in ALL and AML. (DOCX 21 kb) [file 13045_2019_751_MOESM3_ESM.docx]

**Additional file 3: Table S3. Univariate analysis for RI in ALL and AML**

ALL acute lymphoblastic leukemia; Ph+ ALL Philadelphia positive chromosome acute lymphoblastic leukemia; AML acute myeloid leukemia; NA not applicable; CMV cytomegalovirus; NE not executable; BM bone marrow; PB peripheral blood; CR complete remission; ATG anti-thymocyte globulin; PT-Cy post-transplant cyclophosphamide; PS performance status; HCT-CT hematopoietic cell transplantation comorbidity index; MAC myeloablative conditioning regimen; RIC reduced intensity conditioning regimen
