# Supplementary material for: Leukemia relapse following unmanipulated haploidentical transplantation: a risk factor analysis on behalf of the ALWP of the EBMT
Source: J Hematol Oncol. 2019 Jul 4;12:68. doi: 10.1186/s13045-019-0751-4 (PMC6610936; doi:10.1186/s13045-019-0751-4)
Supplement: Supplementary file 4 — Table S4. COX regression for ALL and AML. (DOCX 22 kb) [file 13045_2019_751_MOESM4_ESM.docx]

**Additional file 4: Table S4. COX regression for ALL and AML**

ALL acute lymphoblastic leukemia; AML acute myeloid leukemia; OS overall survival; HR hazard ratio; SCT stem cell transplant; NA not applicable; CR complete remission; HCT-CI hematopoietic cell transplantation comorbidity index; PB peripheral blood; BM bone marrow; CMV cytomegalovirus; PFS progression free survival; NRM non-relapse mortality; MAC myeloablative conditioning regimen; RIC reduced intensity conditioning regimen; aGvHD acute graft-versus-host disease; cGvHD chronic graft-versus-host disease
