# Supplementary material for: Leukemia relapse following unmanipulated haploidentical transplantation: a risk factor analysis on behalf of the ALWP of the EBMT
Source: J Hematol Oncol. 2019 Jul 4;12:68. doi: 10.1186/s13045-019-0751-4 (PMC6610936; doi:10.1186/s13045-019-0751-4)
Supplement: Supplementary file 5 — Table S5. EBMT participating centers. (DOCX 20 kb) [file 13045_2019_751_MOESM5_ESM.docx]

**Additional file 5 : Table S5. EBMT participating centres**

|  | **Patients Numbers** | **Centers** | **Patients Numbers** |
| --- | --- | --- | --- |
| 813 Milano [S Raffaele] | 124 | 250 Saint_Etienne [St Etienne] | 3 |
| 756 Rome [Tor Vergata] | 58 | 277 Lille [H Claude Huriez] | 3 |
| 919 Antalya [Medical Park H] | 51 | 309 Madrid [Jiménez Díaz] | 3 |
| 440 Kocaeli [Anadolu] | 43 | 556 Budapest [National Med Ctr] | 3 |
| 513 Munich [Kl Grosshadern] | 41 | 152 Augsburg [Zentral Kl] | 2 |
| 231 Torino [S. Giovanni (CTO)] | 26 | 169 Ankara [Gazi Univ] | 2 |
| 230 Marseille [Paoli Calmettes] | 20 | 260 Barcelona [SCreu i S Pau] | 2 |
| 931 Suzhou [First Soochow] | 20 | 266 Uppsala [Univ H] | 2 |
| 259 Essen [Univ H] | 19 | 270 Grenoble [H A Michallon] | 2 |
| 808 Dresden [Universitaets Kl] | 19 | 302 Zagreb [Univ H Rebro] | 2 |
| 587 Reggio_Calabria [Centro Trapianti] | 17 | 502 Venezia [SS Giovani e Paolo] | 2 |
| 589 Adana [Baskent Univ] | 14 | 650 Angers [CHRU] | 2 |
| 705 Udine [Univ H] | 14 | 656 Prague [Ist Hematology] | 2 |
| 354 Milano [Trap Mid Osseo] | 13 | 693 Warsaw [Inst Haematology] | 2 |
| 544 Monza [Osp S Gerardo] | 13 | 722 Palma_De_Mallorca [Son Dureta] | 2 |
| 727 Salamanca [H Clinico] | 12 | 861 Potenza [Centro Oncologico] | 2 |
| 163 Piacenza [Osp Civile] | 9 | 925 St._Petersburg [FCHBE] | 2 |
| 261 Geneva [261] | 8 | 281 Patras [Univ H] | 1 |
| 397 Riyadh [King Faisal] | 8 | 284 Birmingham [Heartlands H] | 1 |
| 658 Bergamo [Ospedale, ematol] | 8 | 295 Hannover [Medical Univ] | 1 |
| 307 Rome [Univ S Cuore] | 6 | 304 Firenze [Careggi-Meyer] | 1 |
| 994 Istanbul [Nightingale] | 6 | 305 Torino [Regina Margherita] | 1 |
| 242 Santander [Valdecilla] | 5 | 311 Wiesbaden [Kl Diagnostik] | 1 |
| 524 Heidelberg [Medizinische Kl] | 5 | 345 Haifa [Rambam MCH] | 1 |
| 623 Verona [Policlinico] | 5 | 369 Beirut [American Univ] | 1 |
| 703 Alger [P et M Curie] | 5 | 439 Amman [Al Khalidi] | 1 |
| 141 Brescia [Civili, Adulti] | 4 | 515 Helsinki [Univ Central H] | 1 |
| 209 Leuven [Univ H] | 4 | 624 Toulouse [H Purpan] | 1 |
| 616 Milano [INT] | 4 | 666 Villejuif [Gustave Roussy] | 1 |
| 718 Pilsen [Charles Univ H] | 4 | 671 Lyon [H E Herriot] | 1 |
| 202 Basel [202] | 3 | 726 Liege [University] | 1 |
| 211 Sao_Paulo [H Sirio-Libanes] | 3 | 778 Sheffield [Royal Hallamshire] | 1 |
| 212 Stockholm [Univ H] | 3 | 810 Freiburg [University] | 1 |
