# Supplementary material for: Leukemia relapse following unmanipulated haploidentical transplantation: a risk factor analysis on behalf of the ALWP of the EBMT
Source: J Hematol Oncol. 2019 Jul 4;12:68. doi: 10.1186/s13045-019-0751-4 (PMC6610936; doi:10.1186/s13045-019-0751-4)
Supplement: Supplementary file 5 [file 13045_2019_751_MOESM2_ESM.docx]

Additional file 2: Table S2. Characteristics of relapsed patients

		All patients<br>197		ALL<br>54		AML<br>143	p-value
Patient age		44.54 (range: 18.47-76.08)		30.16 (range 18.48-76.08)		49.18 (range 18.47-72.23)	<0.0001
Year of transplant		2012 (range: 2007-2014)		2012 (range 2007-2014)		2012 (range 2007-2014)	0.4185
Time from transplant to relapse in months		4.91 (range: 0.03-62.77)		4.83 (range 0.03-26.56)		4.94 (range 0.49-62.77)	0.9449
Disease status at transplant	CR1	47 (24%)		14 (26.4%)		33 (23.1%)	0.3856
	CR2+	38 (19.4%)		13 (24.5%)		25 (17.5%)	
	not CR	111 (56.6%)		26 (49.1%)		85 (59.4%)	
Relapse before SCT	NO	94 (47.7%)		19 (35.2%)		75 (52.4%)	0.0451
	YES	103 (52.3%)		35 (64.8%)		68 (47.6%)	
Patient's sex	Female	76 (38.6%)		16 (29.6%)		60 (42%)	0.1551
	Male	121 (61.4%)		38 (70.4%)		83 (58%)	
Donor's sex	Female	85 (43.1%)		22 (40.7%)		63 (44.1%)	0.7965
	Male	112 (56.9%)		32 (59.3%)		80 (55.9%)	
Donor/Host sex	Female to female	32 (16.2%)		7 (13%)		25 (17.5%)	0.3933
	Female to male	53 (26.9%)		15 (27.8%)		38 (26.6%)	
	Male to female	44 (22.3%)		9 (16.7%)		35 (24.5%)	
	Male to male	68 (34.5%)		23 (42.6%)		45 (31.5%)	
Cytogenetics			Ph+	26 (48.1%)	good	6 (4.2%)	
			Ph-	14 (25.9%)	inerm	74 (51.7%)	
			Missing	14 (25.9%)	poor	44 (30.8%)	
					NA/failed	19 (13.3%)	
Conditioning	MAC	95 (48.2%)		38 (70.4%)		57 (39.9%)	0.0002
	RIC	102 (51.8%)		16 (29.6%)		86 (60.1%)	
In vivo T-cell depletion	ATG no PT-Cy	76 (38.6%)		21 (38.9%)		55 (38.5%)	1
	PT-Cy no ATG	121 (61.4%)		33 (61.1%)		88 (61.5%)	
Stem cell source	BM	85 (43.1%)		20 (37%)		65 (45.5%)	0.3666
	PB	112 (56.9%)		34 (63%)		78 (54.5%)	
Sorror HCT-CI		Missing: 15/ Available: 182		Missing: 6/ Available: 48		Missing: 9/ Available: 134	
	HCT-CI 0	73 (40.1%)		28 (58.3%)		45 (33.6%)	0.0107
	HCT-CI 1-2	57 (31.3%)		11 (22.9%)		46 (34.3%)	
	HCT-CI ≥ 3	52 (28.6%)		9 (18.8%)		43 (32.1%)	
Type of relapse	hematological	168 (85.3%)		40 (74.1%)		128 (89.5%)	0.0123
	extramedullary	29 (14.7%)		14 (25.9%)		15 (10.5%)	

CR complete remission; SCT stem cell transplant; NA not applicable; MAC myeloablative conditioning regimen; RIC reduced intensity conditioning regimen; ATG anti-thymocyte globulin; PTCy post-transplant cyclophosphamide ; HCT-CI hematopoietic cell transplantation comorbidity index
